# Durational Differences of Word-Final /s/ Emerge From the Lexicon: Modelling Morpho-Phonetic Effects in Pseudowords With Linear Discriminative Learning

**DOI:** 10.3389/fpsyg.2021.680889

**Published:** 2021-08-09

**Authors:** Dominic Schmitz, Ingo Plag, Dinah Baer-Henney, Simon David Stein

**Affiliations:** ^1^English Language and Linguistics, Heinrich Heine University, Düsseldorf, Germany; ^2^Linguistics and Information Science, Heinrich Heine University, Düsseldorf, Germany

**Keywords:** morphology, speech production, linear discriminative learning, computational modelling, pseudoword paradigm, subphonemic differences

## Abstract

Recent research has shown that seemingly identical suffixes such as word-final /s/ in English show systematic differences in their phonetic realisations. Most recently, durational differences between different types of /s/ have been found to also hold for pseudowords: the duration of /s/ is longest in non-morphemic contexts, shorter with suffixes, and shortest in clitics. At the theoretical level such systematic differences are unexpected and unaccounted for in current theories of speech production. Following a recent approach, we implemented a linear discriminative learning network trained on real word data in order to predict the duration of word-final non-morphemic and plural /s/ in pseudowords using production data by a previous production study. It is demonstrated that the duration of word-final /s/ in pseudowords can be predicted by LDL networks trained on real word data. That is, duration of word-final /s/ in pseudowords can be predicted based on their relations to the lexicon.

## Introduction

Many studies on the acoustic properties of phonologically homophonous elements have shown unexpected effects of their morphological structure on their phonetic realisation. Such effects were shown for seemingly homophonous lexemes (Gahl, 2008; Drager, 2011), for free and bound variants of stems (Kemps et al., 2005a, b), and for prefixes (Ben Hedia and Plag, 2017; Ben Hedia, 2019).

For the level of individual segments, a number of studies have shown that the acoustic realisation of word-final /s/ and /z/ (henceforth S) in English depends on its morphological status and category. Corpus studies (Zimmermann, 2016; Plag et al., 2017) found that non-morphemic word-final S shows longest acoustic durations, followed by suffixes, which in turn are followed by clitics. Experimental studies (Walsh and Parker, 1983; Hsieh et al., 1999; Seyfarth et al., 2017; Plag et al., 2020) confirm durational differences between different types of S. However, their results are mostly not as clear as those by previous corpus studies. That is, only recently a study by Schmitz et al. (2020) on word-final S in pseudowords confirmed the pattern of durational differences found previously only in corpus studies.

Most importantly, none of the aforementioned studies on the matter of word-final S was able to explain found differences on a theoretical level. Traditional models of speech production come with the assumption of having no morphological information in phonetic processing (Levelt et al., 1999; Roelofs and Ferreira, 2019; Turk and Shattuck-Hufnagel, 2020), thus rendering an explanation on the basis of differing morphological categories improbable. Other accounts, e.g., standard feed-forward theories of morphology-phonology interaction (e.g., Chomsky and Halle, 1968; Kiparsky, 1982) or prosodic phonology (e.g., Booij, 1983; Selkirk, 1996; Goad, 1998, 2002), do not offer a satisfying explanation for such durational differences, either.

Only recently, Tomaschek et al. (2019) analysed durational differences between types of S by means of an implementation of naïve discriminative learning (Ramscar and Yarlett, 2007; Ramscar et al., 2010; Baayen et al., 2011). Their results indicate that the duration of a word-final S in English can be sufficiently approximated by considering the support for its morphological function from the word’s sublexical and collocational properties.

This paper continues this line of evidence by making use of the computational model of linear discriminative learning (Baayen et al., 2019b; Chuang et al., 2020), the more advanced successor of naïve discriminative learning. We analyse the durational differences between non-morphemic and plural word-final /s/ found not in real words, but in pseudowords. By using nonce words, we want to rule out potentially confounding effects of the lexical and contextual properties of the individual utterances (e.g., Caselli et al., 2016). Making use of measures derived from this implementation of linear discriminative learning, the present study demonstrates that the effects found by Tomaschek et al. (2019) can be confirmed. Differences in phonetic duration emerge from differences in the strengths of associations between form and meaning.

We proceed as follows. The next section will give an overview on studies on the duration of word-final S, and possibilities and obstacles of theoretical accounts. Section “Introduction to LDL” introduces linear discriminative learning on a theoretical level, while Section “Combining Real Words and Pseudowords in an LDL Implementation” presents the implementation of linear discriminative learning used in the present study. The analysis and results of our study are given in Sections “Analysis” and “Results.” A discussion of the obtained results and a conclusion follow in Section “Discussion.”

## Word-Final /s/ and Its Duration

A number of morphological categories can take the phonological form of /s/ in English, i.e., plural, genitive, genitive plural, third person singular, and the clitics of is, has, and us. In itself, there is nothing in the phonological form of these morphological categories that indicates systematic differences in realisation on the phonetic level between different S morphemes or a non-morphemic S. Yet, a number of studies report on durational differences between different types of S.

Corpus studies on word-final S in English find differences in duration between non-morphemic, suffix, and clitic variants. Zimmermann (2016) on New Zealand English, and Plag et al. (2017) and Tomaschek et al. (2019) on North American English find that non-morphemic S (as in grace, cheese, bus) shows longer durations than plural S and the clitic S of has and is, while plural S in turn shows longer durations than clitic S.

Turning to experimental studies, results are not as consistent. Walsh and Parker (1983) conducted a production experiment with three homophonous word pairs with all words ending in either a non-morphemic or morphemic word-final S. Tested in three different contexts, they find durational differences in two of them. They conclude that morphemic S in English is systematically lengthened by speakers (Walsh and Parker, 1983: 204). However, their conclusion relies on only a small number of 110 observations, a mixture of common and proper nouns as items, and lacks appropriate inferential statistical methods as well as an integration of covariates.

Hsieh et al. (1999) find that plural S is longer than third person singular S in child-directed speech. However, as their data was originally elicited for another study (Swanson and Leonard, 1994), half of all plural items occurred sentence-finally, while almost all third person singular items occurred sentence-medial. Thus, the durational differences found by Hsieh et al. (1999) may be attributed to effects of phrase-final lengthening (e.g., Klatt, 1976; Wightman et al., 1992) rather than to phonetic differences between different types of S.

In another study, Seyfarth et al. (2017) conducted a production experiment on word-final /s/ and /z/ in non-morphemic, plural, and third person singular contexts. Their results indicate that non-morphemic S is shorter than morphemic S. However, they do not find a difference between voiced and voiceless instances, even though previous studies confirm differences dependent on voicing (e.g., Plag et al., 2017). With only six items ending in /s/, but twenty items ending in /z/, it is questionable how meaningful their results on different types of S are.

Comparing affixes, Plag et al. (2020) find that plural and genitive plural S differ in duration. That is, in their study the genitive plural suffix shows a longer duration than the plural suffix.

Most recently, Schmitz et al. (2020) conducted a production experiment on pseudowords carrying either a non-morphemic, plural, or is- or has-clitic S. Their results are in line with those of aforementioned corpus studies. That is, non-morphemic S shows longest S durations, followed by plural S, which in turn is followed by clitic S, while there is no significant durational difference between the two clitics. An overview of the durational differences found in corpus and experimental studies is given in Table 1.

**TABLE 1:** Overview of durational differences of word-final /s/ found in previous studies.

Study	Findings
Zimmermann, 2016; Plag et al., 2017; Tomaschek et al., 2019; Schmitz et al., 2020	Non-morphemic > plural > clitics
Walsh and Parker, 1983	Plural > non-morphemic
Hsieh et al., 1999	Plural > third person singular
Seyfarth et al., 2017	Plural > non-morphemic
Plag et al., 2020	Genitive plural > plural

There is a noteworthy discrepancy between experimental results and the results based on conversational speech data. Results of corpus studies are in line with each other, but they might be flawed due to imbalanced data sets. Experimental studies, on the other hand, often rely on small data sets, and lack phonetic covariates, appropriate statistical methods, or a proper distinction of voiced and voiceless segments. Additionally, previous experimental studies rely on different experimental methods, making their results subject to their pertinent limitations. Another crucial difference between corpus and experimental studies is the use of homophones. While corpus studies take into consideration all words, most experimental studies restrict their data to homophone pairs. This limitation to homophones and the competition between their representations might be a problem of its own as it is unclear how members of such homophone pairs may influence each other in speech production. Lastly, differences in results might also arise due to potentially confounding effects of the lexical properties and contextual effects of the items under investigation.

But even if the direction of durational differences between different types of S is not entirely clear yet, it appears that there are indeed durational differences of some sort. How is one to explain such differences? In standard feed-forward theories of morphology-phonology interaction (e.g., Chomsky and Halle, 1968; Kiparsky, 1982) all types of S, morphemic and non-morphemic, are treated in a similar way. For morphologically complex words, e.g., words ending in a morphological word-final S, a process named “bracket erasure” is said to remove any morphological information. Thus, leaving speech production with no information on the morphology of a complex word (e.g., the plural form cats), rendering its morphological information equal to that of a morphologically simple word ending in a non-morphemic word-final S (e.g., the singular form bus). In such a system, there is nothing that could account for realisational differences between phonologically identical forms of suffixes, clitics, and non-morphemic segments.

A similar distinction of lexical and post-lexical processing is also found in established theories of psycholinguistics. According to models of speech production (e.g., Levelt et al., 1999; Roelofs and Ferreira, 2019), morphemic types of word-final S do not differ in their realisation from non-morphemic instances of word-final S. For a plural form, e.g., cats, the lemma of the lexical concept CAT and a plural specification are retrieved. Then, during morphological encoding, the plural specification is mapped onto the base lemma, i.e., cat, and the plural suffix, < -s >. During phonological encoding, phonemes are selected for the corresponding morphemes, i.e., /k/, /æ/, /t/, and /s/. Finally, the phonemes are syllabified, resulting in a phonological word representation. Such phonological forms are then forwarded and used in speech production. Thus, no information on the morphological origin of particular segments is contained in the phonetic realisation, rendering an explanation on durational differences between types of S on morphological grounds improbable.

**FIGURE 1:**
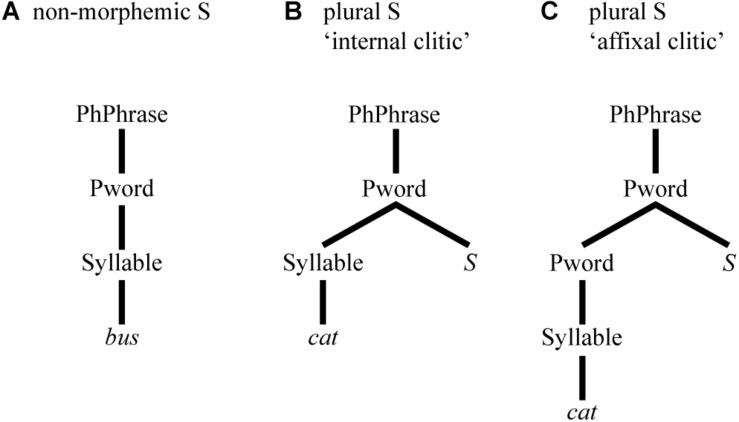
Prosodic configuration for **(A)** non-morphemic and **(B,C)** plural S.

In prosodic phonology (e.g., Booij, 1983), differences in phonetic realisation may arise from the position of sounds in different configurations of prosodic constituency. For instance, different types of word-final S can be analysed as being integrated at different levels of the hierarchical prosodic configuration. In the case of word-final S, different levels co-determine differing degrees of integration of an S to the word it belongs to. Non-morphemic S, uncontroversially, is an integral part of the prosodic word itself (Selkirk, 1996), see (A) of Figure 1. For plural S, Goad (1998) analyses it as an “internal clitic”, see (B), while Goad (2002) analyses it as an “affixal clitic”, see (C).

Thus, the prosodic approach posits a structural prosodic difference between types of S. However, it is not so clear what particular phonetic effects these differences would predict. Most plausibly, a higher degree of integration would correlate with shorter durations, predicting shortest S durations in monomorphemic words. Yet, findings on S duration show the opposite (e.g., Zimmermann, 2016; Plag et al., 2017; Tomaschek et al., 2019; Schmitz et al., 2020), i.e., the duration of non-morphemic S is longest.

An alternative explanation for durational differences between different types of S can be found within the computational modelling framework of naïve discriminate learning (NDL; e.g., Ramscar and Yarlett, 2007; Ramscar et al., 2010; Baayen et al., 2011). NDL is based on simple but powerful principles of discriminative learning theory (Wagner and Rescorla, 1972; Rescorla, 1988), i.e., learning results from exposure to informative relations among events in the individual’s environment. Such events are used to form associations between them, while the associations and their resulting representations are constantly updated on the basis of new experiences. Associations are built between features (“cues”, e.g., biphones) and content lexemes or morphological functions (“outcomes”, e.g., different types of S), which co-occur in events in which the individual is predicting outcomes from cues (Tomaschek et al., 2019: 11). Using the Rescorla-Wagner equations (Rescorla and Wagner, 1972; Wagner and Rescorla, 1972; Rescorla, 1988), relations between cues and outcomes are modelled. That is, the weight of an association, i.e., its strength, increases every time a cue and an outcome co-occur, while it decreases if a cue occurs without the outcome. The result of this process is a continuous recalibration of association strengths, which is a crucial part of discriminative learning.

NDL has been used successfully to model various morphological phenomena, e.g., reaction times in studies on morphological processing (e.g., Baayen et al., 2011; Blevins et al., 2016; see Plag, 2018, chapter 7 for an introduction to NDL in morphological research). For word-final S, Tomaschek et al. (2019) reproduce the differences in duration found by Plag et al. (2017) by means of NDL measures. Their study shows that the duration of different types of S can be approximated by considering the support for these morphological functions from a word’s sublexical and collocational properties. In the NDL network, all words and their diphones within a five word window centred on the target word that contained the S served as cues, and were associated with the morphological functions, which served as outcomes. Two main measurements from this network emerged as predictive for S duration. First, the so-called “activation” as a measure of an outcome’s baseline activation, i.e., of how well an outcome is entrenched in the lexicon. Second, the so-called “activation diversity” as a measure to quantify the extent to which the cues in a given context also support other targets. Taken together, the following pattern for S duration emerges: When the uncertainty about a targeted outcome increases, i.e., the level of “activation” decreases and the level of “activation diversity” increases, the duration of S decreases. In other words: The stronger the support for a morphological function is, both from long-term entrenchment and short-term from the context, the longer its duration.

While NDL implementations apparently offer some form of explanation for different durations of different types of S, they also come with shortcomings and limitations. In NDL, a word’s meaning is defined in terms of the presence or absence of an outcome, i.e., NDL “adopted a stark form of naive realism” (Baayen et al., 2019b: 4) just for computational reasons. That is, NDL takes into account that words tend to have similar forms, but ignores that words are also similar in meaning. Thus, Baayen et al. (2019b) introduced semantic vectors of reals replacing the binarily coded row vectors of the semantic matrix (see Section “The S Matrix: Semantic Vectors”), naming their new implementation linear discriminative learning (LDL) instead of naïve discriminative learning. Outcomes are no longer assumed to be independent, i.e., semantic similarities are now reflected, and networks are mathematically equivalent to linear mappings of matrices, i.e., vector spaces. It is the implementation of such linear discriminative learning that the present paper makes use of for analysing the duration of word-final types of S. Our paper explores whether measures derived from an LDL implementation are predictive of different types of S and their durations. In order to better understand the relation between traditional psycholinguistic variables (such as lexical frequencies, neighbourhood densities, bigram probabilities, morphological category etc.) and LDL measurements we also compare models that use measures derived from an LDL implementation with models that use traditional measures to predict S durations. Finally, we test whether measures derived from an LDL implementation render the specification of morphological structure proper (affix vs. no affix) as predictor variable for S duration unnecessary.

## Introduction to LDL

### Overview

Linear discriminative learning as a computational model implements a discriminative view of learning. In contrast to deep learning models that have multiple hidden layers based on non-linear functions, LDL networks are very simple two-layer networks and are linguistically transparent and interpretable. In LDL, the mental lexicon consists of five high-dimensional numeric matrices, each of which represents a different subsystem: the visual matrix, retina; the auditory matrix, cochlea; the semantic matrix; the speech matrix, speaking; and the spelling matrix, typing. For the current implementation, the semantic and the speech matrix are most important.

With regard to the mappings between vectors, linear mappings are implemented. These mappings are estimated using the linear algebra of multivariate regression. Thus, each mapping is defined by a matrix *A* that transforms the row vectors in a matrix *X* into the row vectors of a matrix *Y*, i.e., *Y* = *XA*. Then, *A* = *X*′*Y*, where *X*′ is the generalised inverse of *X*. We will return to the mapping of matrices in Section “Comprehension and Production,” and refer the interested reader to Baayen et al. (2019b) for an introduction to the mathematical details, as well as to Milin et al. (2017) for a detailed discussion on the restrictions and possibilities of linear mappings.

Another important feature of LDL is its notion of lexomes, i.e., basic semantic units corresponding to words or morphological functions. As outlined in Chuang et al. (2020), lexomes fall into two groups: content lexomes, and inflectional and derivational lexomes. Content lexomes can be morphologically simple or complex forms, i.e., *cat* and *cats*. Inflectional lexomes represent inflectional functions, e.g., number, tense, and aspect. Derivational lexomes represent derivational functions, e.g., morphological categories such as -NESS, -LESS, or UN-. Each lexome is paired with a vector of the aforementioned five subsystems. That is, for the semantic matrix, each lexome is paired with a semantic vector, making each lexome a pointer to a semantic vector on the one hand (Milin et al., 2017), and a location in a high-dimensional space on the other hand. For monomorphemic words, the semantic vector is identical to the semantic vector of the corresponding lexome. That is, the semantic vector of the word *cat*, $\vec{cat}$, is identical to the vector of the lexome CAT. For complex words, the semantic vector is the sum of its corresponding lexome vectors. That is, the semantic vector of the word *cats*, $\vec{cats}$, is the sum of the semantic vectors of the lexomes CAT and PLURAL, $\vec{cat}+\vec{plural}$. The implementation of LDL and the matrices necessary for the present paper are introduced in the subsequent sections. Please refer to https://osf.io/zy7ar/?view_only=ef43a5caf6444270a56074027d7d6482 for the full documentation of the data set, the implementation in R (R Core Team, 2020), and the R script.

#### The S Matrix: Semantic Vectors

The semantic matrix *S* contains semantic vectors of word forms on basis of their corresponding lexomes. That is, the semantic vector $\vec{s}$ in *S* for a simplex word is identical to its corresponding lexome, while the semantic vector $\vec{s}$ in *S* for a cosmplex word is the sum of its corresponding lexomes, e.g., $\vec{apple}+\vec{plural}$ for *apples* (Baayen et al., 2019b). Semantic vectors of lexomes can be derived in different ways (e.g., Landauer and Dumais, 1997; Jones and Mewhort, 2007; Shaoul and Westbury, 2010; Mikolov et al., 2013).

#### The C Matrix: Form Vectors

The present study uses triphones to represent form, as previous studies (Milin et al., 2017; Baayen et al., 2019b; Chuang et al., 2020) have shown that triphones capture the variability of neighbouring phonological information well for English. Triphones are sequences of three phones within a word form. They overlap and can be understood as proxies for phonetic transitions. The cue matrix *C* encodes the forms of words in a binary fashion, giving information on which triphones are part of which word. This is illustrated in (1). In each word’s individual form vector $\vec{c}$, the presence of a triphone is marked with 1, while the absence is marked with 0. The cue vectors of all words of a set of words constitute its *C* matrix. That is, each row in such a *C* matrix represents a word form, while the columns of the respective *C* matrix represent all triphones of its underlying word set.

#### Comprehension and Production

In LDL, comprehension refers to a model that has form vectors as input and semantic vectors as output. We illustrate the *C* matrix of a set of words with a toy lexicon containing the words *cat*, *bus*, and *eel* in (1). Here, the DISC keyboard phonetic alphabet (the “Distinct Single Character” representation introduced by Burnage, 1988) is used for triphone representation. Word boundaries are marked by the # symbol.

$$C\;=\;\begin{array}{c} \;\\ cat\\ bus\\ eel \end{array}\begin{array}{c} \;\#k\{\;k\{t\;\{t\#\;\#bV\;bVs\;Vs\#\;\#il\;il\#\\ \left(\begin{array}{cccccccc} \;1\; & 1\; & 1\; & 0\; & 0\; & 0\; & 0\; & 0\;\\ \;0\; & 0\; & 0\; & 1\; & 1\; & 1\; & 0\; & 0\;\\ \;0\; & 0\; & 0\; & 0\; & 0\; & 0\; & 1\; & 1\; \end{array}\right). \end{array}$$

For the same toy lexicon, suppose that the semantic vectors for these three words are the row vectors of the following *S* matrix:

$$S\;=\;\begin{array}{c} \;\\ cat\\ bus\\ eel \end{array}\begin{array}{c} \;cat\;bus\;eel\\ \left(\begin{array}{ccc} 1.0 & 0.2 & 0.5\\ 0.4 & 1.0 & 0.1\\ 0.2 & 0.3 & 1.0 \end{array}\right). \end{array}$$

To map forms onto meanings we need transformation matrix *F*, such that

CF=S.

The transformation matrix *F* is straightforward to obtain. Let *C*′ denote the Moore-Penrose generalised inverse^1^ of *C*, available in R as the *ginv* function of the MASS package (Venables and Ripley, 2002). Then,

$$F=C^{\prime }S.$$

For the toy lexicon example,

$$F\;=\;\begin{array}{c} \;\\ \#k\{\\ k\{t\\ \{t\#\\ \#bV\\ bVs\\ Vs\#\\ \#il\\ il\# \end{array}\begin{array}{c} \;cat\;bus\;eel\\ \left(\begin{array}{ccc} 0.33 & 0.06 & 0.16\\ 0.33 & 0.06 & 0.16\\ 0.33 & 0.06 & 0.16\\ 0.13 & 0.33 & 0.03\\ 0.13 & 0.33 & 0.03\\ 0.13 & 0.33 & 0.03\\ 0.10 & 0.15 & 0.50\\ 0.10 & 0.15 & 0.50 \end{array}\right), \end{array}$$

with *CF* being exactly equal to *S* in this simple example. That is, taking form vectors as input for the prediction of semantic vectors as output, i.e., solving $\hat{S}=CF$, this toy example correctly predicts 100% of all (three) words’ semantics, i.e., ${\hat{s}}_{i}=s_{i}$. In more complex cases, semantic vectors are only approximately identical, thus, for a word *i* and its predicted semantic vector ${\hat{s}}_{i}$, comprehension is successful if ${\hat{s}}_{i}$ shows the highest correlation with the targeted semantic vector *s_i_* (Baayen et al., 2019b). Following this method, one can report the percentage of comprehension accuracy.

Production as modelled in in LDL takes semantic vectors as input and delivers form vectors as output. Using the same toy lexicon as before, we adapt its *C* matrix, i.e., we borrow the notation by Baayen et al. (2019b) and henceforth call it *T* as is contains the Targeted triphones. For production, the transformation matrix *G* is of interest. Similar to *F* for comprehension, it is straightforward to obtain. Let *S*′ denote the Moore-Penrose generalised inverse of *S*. Then,

$$G=S^{\prime }T.$$

Given *G*, one can then predict the triphone matrix $\hat{T}$ from the semantic matrix *S* by solving

$$\hat{T}=SG.$$

For our toy lexicon example, the *G* transformation matrix is

$$\begin{array}{r} \begin{array}{c} \;\\ \;\\ cat\\ bus\\ eel \end{array}\begin{array}{l} G\;=\;\\ \;\#k\{\;\;k\{t\;\;\{t\#\;\;\#bV\;bVs\;\;Vs\#\;\;\#il\;\;il\#\\ \left(\begin{array}{rrrrrrrr} 1.14 & 1.14 & 1.14 & -0.06 & -0.06 & -0.06 & -0.56 & -0.56\\ -0.44 & -0.44 & -0.44 & 1.05 & 1.05 & 1.05 & 0.12 & 0.12\\ -0.09 & -0.09 & -0.09 & -0.30 & -0.30 & -0.30 & 1.08 & 1.08 \end{array}\right)\;\;. \end{array} \end{array}$$

As this is a toy example, *SG* is identical to *T*. For more complex cases, $\hat{T}$ will not be virtually identical to *T* “but will be an approximation of it that is optimal in the least squares sense” (Baayen et al., 2019b: 21). Triphones with strongest support are expected to be the triphones making up a word’s form. As triphones are not ordered, it is also checked whether the sequence of phones can be constructed correctly. Both, checking triphone support and sequence, are conveniently done by the functions of the WpmWithLdl package (Baayen et al., 2019a). Following this method, one can report the percentage of production accuracy.

**FIGURE 2:**
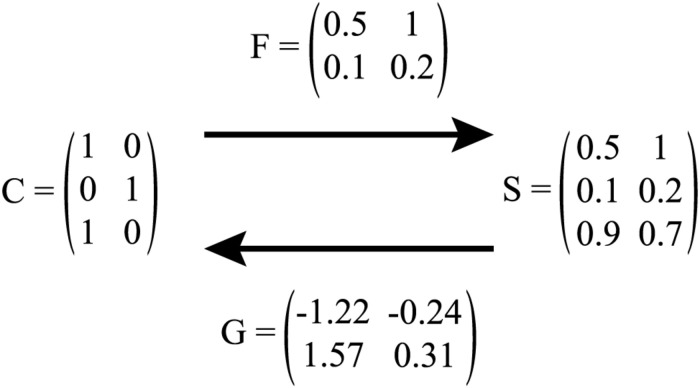
Illustration of mapping between *C* and *S* matrix via *F* (i.e., comprehension), and *S* and *C* matrix via *G* (i.e., production). In production, *C* is referred to as *T*.

Figure 2 summarizes the mapping between form and meaning by the *F* and *G* transformation matrix for comprehension and production modelling.

## Combining Real Words and Pseudowords in an LDL Implementation

### The Semantics of Pseudowords

The present paper follows the implementational basics outlined in Section “Introduction to LDL.” However, as we are interested in /s/ durations in pseudowords (and not in real words), there are a number of complications. The most important complication arises from the widely shared belief that pseudowords do not have meaning. So how can we map form and meaning with forms that have no meaning? In a recent study (Chuang et al., 2020) it was shown that the assumption that pseudowords are bare of meaning is most probably wrong. Due to their formal similarity with existing words, pseudowords resonate with the lexicon. As a result, they may in fact carry meaning. The authors demonstrate that quantitative measures gauging the semantic neighbourhoods of pseudowords predict reaction times of lexical decision and acoustic durations. The present study is inspired by these results and implements a similar architecture. To model resonance of pseudowords with the lexicon, both real words and pseudowords must be included in the networks. The following sections will detail the combined LDL implementation of real words and pseudowords.

### Data Set: Real Words and Pseudowords

The pseudowords and their phonetic realisations that this paper is based on are taken from the study of word-final /s/ production by Schmitz et al. (2020). In their study, participants were given pictures of “alien creatures” and their respective names (which were the target pseudowords), a short explanation of a situation, and a question relevant to the situation which was to be answered aloud. For each participant, pairings of pictures and pseudowords were randomised. That is, each pseudoword was represented by different pictures across participants. By button-press, a question was given to elicit an answer with the pertinent type of S while the context slowly faded out. The fading out of the question forced participants not to rely on the reading-aloud of the given context. In total, 24 pairs of pseudowords were used in that study. Each pseudoword form can act as singular or plural noun, e.g., *glaits* is either realised as singular, i.e., *a glaits*, or as plural, i.e., *two glaits*. Additionally, some pseudowords show a number of different realisations by the participants in the experiment, e.g., *prups* is sometimes produced as /pɹʌps/, and sometimes it is produced as /pɹups/. Thus, not 48 (i.e., 2 × 24) but 78 different phonological forms are included in the pseudoword set. Supplementary Table 1 gives an overview of all pseudowords and their phonological forms.

The second set of words contains real words and their phonetic realisations. Following Chuang et al. (2020) we extracted these words from the MALD corpus (Tucker et al., 2019a). While the MALD corpus contains 26,793 real words, only a subset of 8,285 words is used for a number of reasons. First, some 7,577 words in the corpus contain multiple affixes. As it was unclear how to handle such words, these were excluded. Second, only words for which we have semantic vectors could be used, leading to the exclusion of further 6,828 words. Third, only words with transcriptions available in the CELEX corpus (Baayen et al., 1995) were retained, i.e., there was no transcription available for 818 words. Fourth, 3,285 words showed ambiguities regarding their morphology, e.g., *walks* as a third person singular verb versus the plural of a noun. As huge numbers of words lead to extensive computation times, we decided to exclude such cases as well. The final set of real words contains 6,165 simple and 2,120 complex word forms.

### Cue Matrices

As introduced in Section “The C Matrix: Form Vectors,” cue matrices are coded in binary form, giving information on which triphones are part of which word. For the current implementation, two such cue matrices are created using the WpmWithLdl package’s (Baayen et al., 2019a) *make_cue_matrix* function. First *C*_*rw*_, the real word cue matrix, is created for the set of real words. Then, a second cue matrix, *C*_*pw*_, is created for the set of pseudowords. However, *C*_*pw*_ is a lot smaller than *C*_*rw*_ as there are only 78 phonological forms for pseudowords, but more than 8,000 for real words. Thus, the *C*_*rw*_ is of dimension 8285×7610, while *C*_*pw*_ is of dimension 78×78. We will come back to this issue of differing dimensions in the next section.

### Semantic Matrices

To introduce semantics, i.e., semantic vectors, for the present set of real words, a pre-built semantic matrix *A* from Baayen et al. (2019b) was used. These authors derived semantic vectors based on the TASA corpus (Ivens and Koslin, 1991). For this, words were parsed into their lexomes, i.e., inflected words were represented by their stem and sense-disambiguated labels for their respective inflectional functions. Ambiguous forms, e.g., *walks*, were disambiguated using part of speech tagging (Schmid, 1999). Derived words were assigned a lexome for their stem and a lexome for derivational function. Then, following Baayen et al., 2016 and Milin et al. (2017), Naïve Discriminative Learning (Baayen et al., 2011; Sering et al., 2019) was used to build semantic vectors. The Rescorla-Wagner update rule (Rescorla and Wagner, 1972; Wagner and Rescorla, 1972; Rescorla, 1988) was applied incrementally to the sentences of the TASA corpus. That is, for each sentence the algorithm was given the task to predict the lexomes in that sentence from all lexomes of that sentence. This resulted in a 23562×23562 weight matrix *A*. This matrix lists all lexomes as rows and columns. Thus, for a given lexome at row *i*, the association strengths of this lexome with all other lexomes as given as columns is contained. In this state of the *A* matrix, lexomes predict themselves. Thus, the diagonal of the *A* matrix is set to zero (see Baayen et al., 2019b, for a discussion on this procedure). Lastly, columns which mostly contain zeros, i.e., no information, and show small variances (σ < 3.4 × 10^−8^) are removed. The resulting *A* matrix is of dimension 23,562 × 5030. Following the method outlined in Section “The S Matrix: Semantic Vectors,” a semantic matrix for real words *S*_*rw*_ can be constructed based on *A*. That is, the semantic vector $\vec{s}$ in *S*_*rw*_ for a simplex word is identical to its corresponding lexome, while the semantic vector $\vec{s}$ in *S*_*rw*_ for a complex word is the sum of its corresponding lexomes. That is, the semantic vector of *apple* is $\vec{apple}$, while the semantic vector of *apples* is the sum of the vectors of the lexomes APPLE and PLURAL, i.e., $\vec{apples}=\vec{apple}+\vec{plural}$. As a set of real words is used, *S*_*rw*_ contains only semantic vectors for this set of real words (instead of, e.g., all word forms of the TASA corpus). The final real word semantic matrix *S*_*rw*_ is of dimension 8285 × 5487.

While this procedure is rather straightforward, the creation of a pseudoword semantic matrix *S*_*pw*_ is not. Due to the nature of pseudowords, their lexomes are not contained within any corpus or our *A* matrix, for that matter. Instead, one can estimate a pseudoword’s semantic content by utilising the semantic and phonological information on real words, i.e., their *C* and *S* matrix (Chuang et al., 2020). That is, the same transformation matrix *F* that is used for mapping real word cues onto predicted real word meanings (see Section “Comprehension and Production”) can be used to map pseudoword cues onto their estimated semantics. That is, one must first solve

F=C′rwSrw

to obtain *F*. Then, one can make use of the pseudoword cue matrix *C*_*pw*_, and estimate pseudoword semantics, as

$$S_{pw}=C_{pw}F,$$

with *S*_*pw*_ denoting the originally estimated semantic matrix for pseudowords. In this semantic matrix, pseudowords of identical segmental makeup show identical semantics as semantics are calculated only based on triphone occurrence, i.e., the semantics of *pleeps*_*singular*_ is identical to the semantics of *pleeps*_*plural*_. To differentiate between singular and plural pseudowords, the semantic vector of the PLURAL lexome is added to all plural pseudowords in the S matrix. Similarly, the semantic vectors of ALIEN and CREATURE are added to all pseudoword semantic vectors as participants in the original production experiment were told that pseudowords describe alien creatures. As explained in Section “Model B: LDL Measures and Affix Specification,” the pairing of the pictures with pseudowords representing the alien creature was randomised during the experiment by Schmitz et al. (2020). A pertinent pseudoword thus only contains the semantics of “alien creature” as a constant part of its own semantics, while other factors such as appearance, e.g., colour, shape, or number of eyes, differ across participants. We can assume that in the course of the experiment, participants gradually came to realize that the looks of these alien creatures, i.e., colour, shape, etc., are not relevant to their label names. Thus participants were just aware of the fact that these are all alien creatures, without paying much attention to their individual features. Please see the aforementioned complementary material for a detailed implementation.

### Comprehension and Production

Pseudoword comprehension and production are not computed and evaluated in isolation but in combination with real words, simulating a real person’s lexicon in a pseudoword comprehension and production situation, respectively. For this, we created a cue matrix *C*_*comb*_ based on a combined set of words, containing all aforementioned real words and pseudowords. In total, 8440 word forms are part of this set of words. A combined semantic matrix *S*_*comb*_ is created by attaching *S*_*pw*_ to *S*_*rw*_, and reordering its rows to reflect the same order of words as found in *C*_*comb*_.

Then, using the functions of the WpmWithLdl package (Baayen et al., 2019a) in R, a comprehension model is trained and checked for accuracy. That is, taking form vectors as input for the prediction of semantic vectors of output, ${\hat{S}}_{comb}=C_{comb}F$ is solved. Comprehension is successfully modelled for a word *i* if its predicted semantic vector ${\hat{s}}_{i}$ is most highly correlated with its targeted semantic vector *s_i_*. This is true for 74.41% of cases (i.e., 6,165 word forms) in our comprehension model. In total, 25.59% of cases (i.e., 2,120 word forms) are incorrectly predicted, with 1,912 simple and 208 complex word forms. None of the incorrectly predicted word forms is a pseudoword.

Similarly, a production model is trained and checked for accuracy using functions of the aforementioned R package. Thus, semantic vectors are provided as input to predict form vectors as output, i.e., to solve ${\hat{T}}_{comb}=S_{comb}G$. Production is successfully modelled for a word *i* if its predicted triphones are those triphones present in its targeted cue vector in the correct sequence (possible sequences of triphones will be referred to below as “paths”). This is true for 97.3% of cases (i.e., 8,061 word forms) in our production model. In total, 2.7% of cases (i.e., 224 word forms) are incorrectly predicted, with 98 simple and 126 complex word forms. None of the incorrectly predicted word forms is a pseudoword.

### Measures

In order to explore the potential of different measures emerging from the network to predict phonetic duration, we extracted a whole range of measures, based on the measures introduced by the WpmWithLdl package (Baayen et al., 2019a) and by Chuang et al. (2020). Please see the Supplementary Material for exploratory analyses of individual measures.

In the following, we first describe the semantic measures before we turn to the phonetic measures.

L1NORM and L2NORM: The L1NORM is the sum of the absolute values of vector elements of a given word’s predicted semantic vector $\hat{s}$, i.e., its city-block distance. The L2NORM is the square root of the sum of the squared values of a given word’s predicted vector $\hat{s}$, i.e., its Euclidian distance. For both variables, higher values imply more strong links to many other lexomes. Thus, both measures may be interpreted as semantic activation diversity.

DENSITY: For DENSITY, the correlation values of a word’s predicted semantic vector $\hat{s}$ and its eight nearest neighbours’ semantic vectors *s*_*n*1_⋯*s*_*n*8_ are taken into consideration. The mean of these eight correlation values describes DENSITY, with higher values indicating a denser semantic neighbourhood.

ALC: The Average Lexical Correlation, ALC, is the mean value of all correlation values of a pseudoword’s estimated semantic vector as contained in *S*_*pw*_ with each of the real word semantic vectors as contained in *S*_*rw*_. Higher ALC values indicate that a pseudoword’s semantics are part of a denser semantic neighbourhood. Thus, ALC may be interpreted as a measure of semantic activation diversity for pseudowords.

EDNN: This variable describes the Euclidian Distance between a pseudoword’s estimated semantic vector *s* and its Nearest semantic real word or pseudoword Neighbour. Thus, higher values indicate a larger distance to the nearest semantic neighbour. EDNN may be regarded as a measure of semantic neighbourhood density.

NNC: The Nearest Neighbour Correlation is computed by taking a pseudoword’s estimated semantic vector as given in *S*_*pw*_ and checking it for the highest correlation value against all real word semantic vectors as given in *S*_*rw*_. This highest correlation value is taken as NNC value. Thus, higher values indicate that a pseudoword is semantically close to a real word. Additionally, one can tell which real word a pseudoword’s semantics are closest to. This measure may be interpreted as a measure of similarity between nonce and real words, indicating the co-activation of a real word when confronted with a pseudoword.

SUPPORT: This measure describes the amount of support the word-final triphone (i.e., fs#, ks#, ps#, ts#) obtains for each pseudoword. The value of SUPPORT is extracted from $\hat{T}$. Higher values of this variable indicate a higher semantic support for the word-final triphone which includes the segment of interest, i.e., word-final S.

PATH_COUNTS: PATH_COUNTS describes the number of paths, i.e., possible sequences of triphones, detected for the production of a word by the production model. PATH_COUNTS may be interpreted as a measure of phonological activation diversity, as higher values indicate the existence of multiple candidates (and thus paths) in production.

PATH_SUM: PATH_SUM describes the summed support of paths for a predicted form. PATH_SUM may be interpreted as a measure of phonological certainty, with higher values indicating a higher certainty in the candidate form.

PATH_ENTROPIES: PATH_ENTROPIES contains the Shannon entropy values which are calculated over the path supports of the predicted form in $\hat{T}$. Thus, PATH_ENTROPIES may be interpreted as a measure of phonological uncertainty, with higher values indicating a higher level of disorder, i.e., uncertainty.

ALDC: The Average Levenshtein Distance of all Candidate productions, ALDC, is the mean of all Levenshtein distances of a word and its candidate forms. That is, for a word with only one candidate form, the Levenshtein distance between that word and its candidate form is its ALDC. For words with multiple candidates, the mean of the individual Levenshtein distances between candidates and targeted form constitutes the ALDC. Thus, higher values indicate that a word’s candidate forms are very different from the intended pronunciation. ALDC may be interpreted as a measure of phonological neighbourhood density as it takes into account real word neighbourhoods for pseudowords, i.e., large values indicate sparse real word neighbourhoods.

## Analysis

The data set by Schmitz et al. (2020) contains non-morphemic, plural, or clitic word-final S as final segment of a pseudoword. As our LDL implementation does not include information on clitics, we only consider durational data on non-morphemic and plural S for the present study. A subset of 666 data points remains, with 303 observations with non-morphemic S and 363 observations with plural S. Due to some variable pronunciations requiring triphones not included in our LDL implementation, 13 data points had to be excluded, resulting in a final data set with non-morphemic and plural S durations of 653 data points, i.e., 300 entries on non-morphemic S and 353 entries on plural S.

### Covariates

Besides the aforementioned variables extracted and computed from the LDL implementation itself (see Section “Measures”), the following covariates, adopted from previous analyses of word-final S (e.g., Plag et al., 2017; Tomaschek et al., 2019; Schmitz et al., 2020), are included in the analysis. The main reason for this is to allow us to compare the performance of these predictors with the performance of LDL predictors. LDL measures often correlate with traditional measures (such as lexical frequencies, transitional probabilities, or neighborhood densities), but the traditional measures have no clear correlating mechanisms in learning or processing.

There are, however, also covariates that do not tap into lexical properties, but that control for other influences, such as speech rate, the speaker, gender, the order of stimuli in an experiment, etc. These will be referred to as “non-lexical covariates” and they will also be included in our regression models.

AFFIX: This binary variable indicates whether a word contains an affix, i.e., whether the pertinent pseudoword is a singular or plural form. It takes the value NM for pseudowords without affix, and PL for pseudowords with affix.

SPEAKINGRATE: Analysing durational data, speech rate is a self-evident variable to consider. As speech rate is no inherent part of any LDL measure, we calculated speaking rate as the number of syllables in an utterance divided by the duration of the utterance (e.g., Tomaschek et al., 2019; Schmitz et al., 2020). This was done automatically using a script in Praat (de Jong and Wempe, 2008; Boersma and Weenink, 2019).

BASEDURLOG: Base duration was taken as a more local measure of speech rate (e.g., Plag et al., 2017, 2020; Schmitz et al., 2020). Here, the term “base” refers to the string of segments preceding the word-final S, for both non-morphemic and morphemic pseudowords. Base duration was then log-transformed to achieve a closer to normal distribution.

PAUSEBIN: To account for final-lengthening effects, stretches of silence between the offset of the word-final S and the onset of the following word were measured. Silence of 50 ms and above was considered as pause (Lee and Oh, 1999; Krivokapić, 2007). In order to make sure that closures of following plosives were not mistaken for pauses, their average closure duration (see Yao, 2007) was subtracted of the pertinent measured silence. Following the results by Schmitz et al. (2020), pause information was included as binary variable with the values PAUSE / NO PAUSE.

DISC: As some pseudowords were produced with multiple pronunciations, their transcription was incorporated as a categorical variable. This variable is called DISC after the DISC keyboard phonetic alphabet (Burnage, 1988).

BIPHONEPROBSUMBIN: The summed biphone probability for each pseudoword and its phonological variants is included as the binary variable BIPHONEPROBSUMBIN. It was calculated using the Phonotactic Probability Calculator (Vitevitch and Luce, 2004). The rationale for this variable is that more probable biphones should lead to shorter durations (e.g., Schmitz et al., 2020).

LIST & SLIDENUMBER: To account for priming effects, the list number (1–12) and the point of occurrence during the original experiment by Schmitz et al. (2020) are included.

PREC: To account for potential effects of the consonant preceding the word-final S (Umeda, 1977), it is included as PREC variable (similar to e.g., Tomaschek et al., 2019).

BIPHONEPROB: The probability of the final biphones /fs/, /ks/, /ps/ and /ts/ in monomorphemic words is included as covariate to account for potential effects of phonotactics (see Schmitz et al., 2020, for a detailed explanation).

FOLTYPE: As the segment following the word-final S is no part of the individual pseudoword, it is also not considered in LDL measures. Thus, the covariate FOLTYPE is introduced (similar to e.g., Tomaschek et al., 2019), coding the following segment by its segmental class (i.e., approximant APP for *listen*, fricative F for *find*, nasal N for *know*, plosive P for *cook*, and vowel V for *eat*), to account for potential effects of the following word (Klatt, 1976; Umeda, 1977).

SPEAKER, GENDER, AGE, LOCATION and MONOMULTILINGUAL: SPEAKER ID was included to account for general inter-speaker differences in production. GENDER, AGE, and LOCATION, i.e., the place in which the pertinent participant spent the bigger part of their life, were included as well. Additionally, participants who were early bilinguals were categorised as multilingual, while all other participants were categorised as monolingual in MONOMULTILINGUAL.

REAL: Some of the pseudowords in Schmitz et al.’s data set have an orthographically different, but phonologically identical real word counterpart. We introduced the variable REAL to control for this potential confound. This variable is TRUE for pseudowords with such a real word counterpart, and FALSE for those without. We considered the following real words as counterparts as given in Schmitz et al. (2020): *glits* corresponds to *glitz*, *glaiks* corresponds to *Gleicks*, *glifs* corresponds to *glyphs*, and *pleets* corresponds to *pleats*.

All of the following analyses make use of the following non-lexical covariates: BASEDURLOG, SPEAKINGRATE, SLIDENUMBER, and PAUSEBIN as variables concerning speech rate and continuity, PREC and FOLTYPE accounting for coarticulatory effects, LIST taking into consideration potential priming effects, MONOMULTILINGUAL, GENDER, LOCATION, AGE, and SPEAKER to account for speaker-individual differences, and REAL to include potential effects of real word counterparts.

### Modelling Strategy

We devised three kinds of model: First, a baseline model with the traditional predictor variables (plus the non-lexical covariates). Second, a model with LDL predictors that also includes AFFIX as a covariate (plus the non-lexical covariates). Third, a model that contains only the LDL predictors (plus the non-lexical covariates).

The three kinds of model will allow us to answer our research questions. Recall that our ultimate goal is to understand how systematic durational differences emerge between words of different, but homophonous morphological categories. Traditional lexical variables are predictive but cannot explain how morphology can make its way into durational differences. But these models can show that such differences exist by looking at the effect of the variable AFFIX. This is our baseline model. As an alternative we implement a model that uses LDL measures. If these measures are predictive, they offer an explanation of the morphologically-induced phonetic differences: they emerge as a by-product of the association of form and meaning in the mental lexicon, and this association is the outcome of discriminative learning. By having a model that also includes AFFIX as an additional predictor, we can see whether the LDL measures completely capture the morphological effect, or whether there is a residue of morphological information that is predictive of duration but is still not captured by the LDL measures.

### Model A: Traditional Measures

This model is meant to resemble those in previous studies on word-final S duration (e.g., Plag et al., 2017; Schmitz et al., 2020). Thus, we make use of similar variables: AFFIX, BIPHONEPROBSUMBIN, and BIPHONEPROB, as well as those control variables included in all analyses of this paper. None of these covariates showed high correlation coefficients. Hence, no cautionary measures regarding collinearity were taken before an initial full model was constructed. The model selection process proceeded as explained in section “Model B: LDL Measures and Affix Specification.” That is, non-significant variables were excluded in a controlled step-wise fashion.

Then, variance inflation factors were checked. The covariates BIPHONEPROB and PREC showed high VIF values (i.e., 46.53 and 46.88, respectively), indicating potential overfitting of the model (e.g., Zuur et al., 2010; Fox and Weisberg, 2019). Consequently, PREC was removed from the model as it showed the highest VIF value, following the procedure described by Zuur et al. (2010). Re-fitting the model without PREC and re-checking the new variance inflation factor values revealed only non-problematic values.

Finally, the resulting model’s residuals were trimmed (e.g., Baayen and Milin, 2010). Data points with residuals larger than 2.5 standard deviations were removed, ensuring a satisfactory distribution of residuals. This procedure led to a loss of 4 data points, i.e., 0.61% of all data points. An overview of all variables used in the initial model is given in Supplementary Table 2.

### Model B: LDL Measures and Affix Specification

This model makes use of all LDL measures as well as of the AFFIX variable. Additionally, the non-lexical covariates are included. One issue to address when considering a model with such a multitude of variables is collinearity (e.g., Baayen, 2008; Tomaschek et al., 2018). To avoid collinearity related problems later on, all variables were tested for correlation using the languageR package (Baayen and Shafaei-Bajestan, 2019). This correlation check resulted in eight correlation coefficients indicating a high degree of correlation, for which we assume the threshold to be |*rho*| ≥ 0.5. The pairs of correlated covariates as well as their correlation coefficients are given in Table 2.

**TABLE 2:** Correlated variables and their correlation coefficients.

variables		rho	variables		rho
L1NORM	L2NORM	0.98	AFFIX	NNC	–0.89
PATH_COUNTS	PATH_ENTROPIES	0.95	PATH_COUNTS	SUPPORT	–0.65
PATH_COUNTS	ALDC	0.89	PATH_SUM	SUPPORT	0.73
PATH_ENTROPIES	ALDC	0.90	PATH_ENTROPIES	SUPPORT	–0.63

Due to the high number of correlated variables, we opted for a principal component analysis (PCA; e.g., Venables and Ripley, 2002; Baayen, 2008; Tomaschek et al., 2018) to address collinearity issues. In a PCA, the dimensionality of the data is reduced by transforming the included variables into principal components. These transformations result in linear combinations of the predictors that are orthogonal to each other. Thus, the resulting principal components are not correlated.

The PCA was carried out using the *PCAmix* function of the PCAmixdata package (Chavent et al., 2017) in R, allowing the simultaneous integration of continuous and discrete variables. All variables given in Table 2 were included in the computation of the principal component analysis, which yields nine principal components. The next step of the PCA is to determine how many of these principal components are meaningful and thus should be retained for further use. For this decision, we followed several rules of thumb (e.g., O’Rourke et al., 2005; Baayen, 2008). First, any component that displays an Eigenvalue greater than 1 accounts for a greater amount of variance than had been contributed by one variable. Such a component is therefore potentially meaningful. Second, one should retain enough components so that the cumulative percent of variance explained is equal to some minimal value. Following other implementations of principal component analyses, we aim at a value of 80% (e.g., O’Rourke et al., 2005). Third, only interpretable components are to be retained. That is, each component is made up of loadings, i.e., parts of the variables included in the PCA’s computation represented by correlation coefficient values. If none of these variables is strongly represented in a component, the interpretability of that component is extremely low, rendering the component of small interest for further analyses. Following these three criteria, we find that the first three of the principal components show an Eigenvalue of one or higher. Also, the first three components account for 84% of variance. Considering the third criterion, all three components are strongly correlated with input variables. We therefore retain components 1 to 3 for further analysis, all of which show an Eigenvalue greater than 1, account for more than eighty percent of variance, and contain strong representations of variables in their loadings.^2^ But what do these principal components mean? The highest loadings of the principal components, i.e., the correlation of the original variables to the pertinent component, are given in Table 3.

**TABLE 3:** Loadings of original predictor variables in the three retained principal components of the first principal component analysis.

	Component1	Component2	Component3
L1NORM		0.397	0.348
L2NORM		0.405	0.363
PATH_COUNTS	0.813		
PATH_ENTROPIES	0.828		
PATH_SUM	–0.430		
ALDC	0.710		
NNC		0.698	
SUPPORT	–0.650		
AFFIX		0.421	0.517

COMPONENT1 is most strongly positively correlated with PATH_COUNTS, PATH_ENTROPIES, and ALDC, while it is most strongly negatively correlated with PATH_SUM and SUPPORT. For PATH_COUNTS, higher values indicate the existence of multiple candidates (and thus paths) in production. It thus functions as an indicator of phonological uncertainty. Values of PATH_ENTROPIES relate to the level of uncertainty concerning the path supports of the predicted candidate form, with higher values indicating a higher level of uncertainty. For ALDC, higher values mean that a word’s candidate forms are very different from the intended pronunciation, indicating uncertainty in production. PATH_SUM describes the summed support of paths for a predicted form, with higher values indicating a higher certainty in the candidate form. Higher values for SUPPORT suggest more certainty in the choice of the word-final triphone. COMPONENT1 can thus be described as a dimension that represents phonological or articulatory certainty.

COMPONENT2 is most strongly correlated with L1NORM, L2NORM, NNC, and AFFIX. L1NORM and L2NORM both imply more strong links to many other lexomes with higher values indicating a higher semantic activation diversity. Higher values of NNC suggest a close real word neighbour, which leads to higher levels of co-activation of that real word when confronted with the pseudoword, also leading to higher semantic activation diversity. As for AFFIX, COMPONENT2 is positively correlated with the presence of non-morphemic S data points.

COMPONENT3 is similar to COMPONENT2 as it is also strongly correlated with L1NORM, L2NORM, and AFFIX. Again, for L1NORM and L2NORM higher values indicate higher semantic activation diversity. AFFIX is positively correlated for plural S data points. We will come back to the interpretation of this correlation in Section “Model B: LDL Measures and AFFIX Specification.”

In a next step, models were fitted using linear mixed-effects regression in R (R Core Team, 2020) using RStudio (RStudio Team, 2021) and as implemented by lme4 (Bates et al., 2015), lmerTest (Kuznetsova et al., 2017), and LMERConvenienceFunctions (Tremblay and Ransijn, 2020) to analyse the data on non-morphemic and plural S duration. The dependent variable, duration of S, was log-transformed following standard procedures to reduce the potentially harmful effect of skewed distributions in linear regression models (e.g., Winter, 2019). The name of this variable is SDURLOG.

Following the standard backward step-wise selection process for model selection (e.g., Baayen, 2008), a first model containing all remaining variables is created. That is, COMPONENT1, COMPONENT2, COMPONENT3, DENSITY, ALC, EDNN, BASEDURLOG, SPEAKINGRATE, PAUSEBIN, FOLTYPE, PREC, and REAL were included as fixed effects. The remaining variables, GENDER, LOCATION, MONOMULTILINGUAL, AGE, LIST, and SPEAKER, are included as random intercepts.

This full model was then continuously reduced through step-wise exclusion of non-significant variables. That is, a variable was considered as significant if it passed all of three tests. First, its F-value in the pertinent model had to yield a value below −2 or above 2. Second, the AIC value, i.e., the Akaike information criterion value, of the model including the variable had to be lower than the AIC value of a comparable model without the pertinent variable. Third, the results of log-likelihood tests comparing the model with to a model without the pertinent variable had to yield a p-value below the 0.05 threshold, thus indicating a significant improvement of model fit. This process was verified using the *step* function of R, which resulted in an identical model.

Then, variance inflation factors (VIFs) were computed. Predictors showing variance inflation factor values equal or greater than 3 are to be excluded due to the high risk of introducing multicollinearity and thus overfitting of the model (e.g., Zuur et al., 2010). For the present model, all variance inflation factor values are below 3.

Finally, the resulting model needed trimming of its residuals (e.g., Baayen and Milin, 2010). Data points with residuals larger than 2.5 standard deviations were removed to ensure a more satisfactory residual distribution. This procedure resulted in a loss of six data points (0.92%). An overview of all variables used in the initial model and their distribution is given in Supplementary Table 2.

### Model C: LDL Measures Only

This model uses all LDL measures but does not incorporate the AFFIX covariate. As in the previous model, there was a high number of highly correlated variables (see Table 2 with the exception of the correlation of AFFIX and NNC, as AFFIX is not included in this analysis). We therefore again computed a principal component analysis, following the procedure outlined in Section “Model B: LDL Measures and Affix Specification.” Following the first two criteria, we find that two principal components are to be retained. However, considering the third criterion, we find that the two components are not readily interpretable as they show relatively high positive or negative correlations with all or almost all variables, without indicating a clearly discernible dimension underlying the patterns of correlations. We therefore turned to another procedure to reduce collinearity issues.

For each set of variables with a correlation of |*rho*| > 0.5, models containing only the pertinent variable and a random intercept for subject are fitted and compared. Using log-likelihood tests for model comparison, the variable contained in a significantly better fit model is retained while those variables highly correlated with it are no longer used. In case of a non-significant difference, the variable of the model with the lower AIC value is retained. This procedure leads to the exclusion of L2NORM, PATH_COUNTS, PATH_ENTROPIES, and PATH_SUM.

Linear mixed-effects regression models were fitted according to the procedure given in Section “Model B: LDL Measures and Affix Specification.” That is, an initial full model was fitted with the following variables: L1NORM, ALDC, SUPPORT, DENSITY, ALC, EDNN, NNC, BASEDURLOG, SPEAKINGRATE, PAUSEBIN, FOLTYPE, PREC and REAL. As for random effects, random intercepts for GENDER, LOCATION, MONOMULTILINGUAL, AGE, LIST, and SPEAKER were included.

This full model was then continuously reduced through step-wise exclusion of non-significant variables, following the aforementioned criteria. Then, variance inflation factors were computed, resulting only in non-problematic values (e.g., Zuur et al., 2010). Finally, the resulting model needed trimming of its residuals (e.g., Baayen and Milin, 2010). That is, data points with residuals larger than 2.5 standard deviations were removed, ensuring a more satisfactory residual distribution. This procedure led to a loss of 8 data points, i.e., 1.2% of all data points. An overview of all variables used in the initial model and their distribution is given in Supplementary Table 2.

## Results

### Model A: Traditional Measures

The final model of traditional measures includes main effects of the following variables: type of S (AFFIX), speaking rate (SPEAKINGRATE), log-transformed base duration (BASEDURLOG), pause (PAUSEBIN), the summed biphone probability (BIPHONEPROBSUMBIN), and following segmental type (FOLTYPE). As for random effects, random intercepts for SPEAKER and random slopes for AFFIX are included. The p-values of the analysis of variance of the final model are given in Table 4.

**TABLE 4:** *p*-values of fixed effects in the final “traditional” model, fitted to the log-transformed durations of S.

	Sum Sq	Mean Sq	NumDF	DenDF	F.value	Pr (> F)
AFFIX	0.711	0.711	1	37.90	13.845	0.001
SPEAKINGRATE	0.163	0.163	1	604.07	3.165	0.076
BASEDURLOG	6.278	6.278	1	572.80	122.247	0.000
PAUSEBIN	5.430	5.430	1	635.92	105.722	0.000
BIPHONEPROBSUMBIN	0.646	0.646	1	596.28	12.580	0.000
FOLTYPE	2.199	0.550	4	605.15	10.703	0.000

The marginal R-squared value of the model is 0.43, i.e., fixed effects explain 43% of variation in the data. Taking random effects into account as well, the conditional R-squared value is 0.62. That is, the model explains 62% of data variation in total (see Nakagawa et al., 2017, for details on marginal and conditional R-squared computation). Both R-squared values were computed using the MuMIn package (Barton, 2020). The R-squared values are similar to the values found by Schmitz et al. (2020) on their complete data set.

The estimates of the final model and their p-values are given in Table 5. The reference levels for the categorical predictors are: for AFFIX it is NM, for PAUSEBIN it is no-pause, for BIPHONEPROBSUMBIN it is high, and for FOLTYPE it is APP.

**TABLE 5:** Fixed-effect coefficients and *p*-values as computed by the final “traditional” model (mixed-effects model fitted to the log-transformed duration of S).

	Estimate	Std. Error	*df*	*t*-value	Pre (> | t|)
(Intercept)	−1.202	0.083	407.927	−14.520	0.000
AFFIXPL	−0.087	0.023	37.896	−3.721	0.001
SPEAKINGRATE	−0.022	0.012	604.072	−1.779	0.076
BASEDURLOG	0.635	0.057	572.805	11.057	0.000
PAUSEBINPAUSE	0.234	0.023	635.917	10.282	0.000
BIPHONEPROBSUMBINlow	−0.076	0.021	596.279	−3.547	0.000
FOLTYPEF	−0.001	0.073	610.436	−0.007	0.994
FOLTYPEN	−0.004	0.028	600.528	−0.134	0.893
FOLTYPEP	−0.027	0.025	599.182	−1.107	0.269
FOLTYPEV	−0.145	0.025	610.241	−5.852	0.000

The predictor strength of individual covariates was checked by taking the final model as template. For each predictor variable, a model was fitted lacking the particular variable. This resulted in seven models, each lacking a different predictor. Then, R-squared values were computed for these models and finally compared. The variable leading to the highest decrease in R-squared value as compared to the final model is thus the variable showing the highest predictor strength. The results of this comparison are reflected in the hierarchy given in (1). The decrease in R-squared is greatest when removing BASEDURLOG, followed by PAUSEBIN, and so forth. The resulting order is identical to the one found by Schmitz et al. (2020) for the complete data set.

(1) baseDurLog > > pauseBin > > Affix > > folType > > speakingRate > > biphoneProbSumBin

### Model B: LDL Measures and AFFIX Specification

In the final model including LDL measures as well as the AFFIX covariate as parts of the individual components resulting from the principal component analysis, and fitted according to the procedure described in Section “Model B: LDL Measures and Affix Specification,” we find main effects of the first principal component (COMPONENT1), the third principal component (COMPONENT3), DENSITY, ALC, base duration (BASEDURLOG), following pause (PAUSEBIN), following segmental type (FOLTYPE), and preceding consonant (PREC). Regarding random effects, only a SPEAKER-specific random intercept turns out to significantly improve model fit. The p-values of the analysis of variance of the final model are given in Table 6.

**TABLE 6:** *p*-values of fixed effects in the final “LDL measures and Affix” model, fitted to the log-transformed durations of S.

	Sum Sq	Mean Sq	NumDF	DenDF	F.value	Pr (> F)
COMPONENT1	0.376	0.376	1	618.06	6.970	0.008
COMPONENT3	1.340	1.340	1	627.71	24.819	0.000
BASEDURLOG	6.751	6.751	1	620.55	125.080	0.000
PAUSEBIN	5.805	5.805	1	642.19	107.568	0.000
FOLTYPE	2.093	0.523	4	617.98	9.695	0.000
PREC	0.702	0.234	3	615.33	4.334	0.005
DENSITY	0.219	0.219	1	621.79	4.067	0.044
ALC	0.293	0.293	1	623.25	5.425	0.020

The marginal R-squared value of the final model is 0.42, thus fixed effects explain 42% of the variation in our data. The conditional R-squared value of the final model is 0.60, that is fixed and random effects taken together explain 60% of variation.

The estimates of the final model and their p-values are given in Table 7. The reference levels for the categorical predictors are: for PAUSEBIN it is no-pause, for FOLTYPE it is APP, and for PREC it is f.

**TABLE 7:** Fixed-effect coefficients and *p*-values as computed by the final “LDL measures and Affix” model (mixed-effects model fitted to the log-transformed duration of S).

	Estimate	Std. Error	*df*	*t*-value	Pre (> | t|)
(Intercept)	−1.106	0.124	635.215	−8.952	0.000
COMPONENT1	0.014	0.005	618.057	2.640	0.008
COMPONENT3	−0.041	0.008	627.708	−4.982	0.000
BASEDURLOG	0.652	0.058	620.548	11.184	0.000
PAUSEBINpause	0.237	0.023	642.193	10.371	0.000
FOLTYPEF	−0.014	0.075	621.463	−0.180	0.857
FOLTYPEN	−0.006	0.029	614.760	−0.198	0.843
FOLTYPEP	−0.028	0.025	615.172	−1.126	0.261
FOLTYPEV	−0.141	0.025	620.352	−5.612	0.000
PRECk	−0.023	0.027	614.436	−0.835	0.404
PRECp	−0.040	0.027	614.491	−1.475	0.141
PRECt	−0.095	0.028	615.916	−3.414	0.001
DENSITY	−0.241	0.119	621.790	−2.017	0.044
ALC	−5.302	2.277	623.246	−2.329	0.020

Similar to Section “Model B: LDL Measures and AFFIX Specification,” the predictor strength of individual covariates was checked by taking the final model as template. For each predictor variable, a model was fitted lacking the pertinent variable. This resulted in seven models, each missing a different covariate. Then, marginal R-squared values were computed and compared. The model showing the lowest of these values in turn missed the covariate with the highest predictor strength. The result of this procedure is reflected in the hierarchy in (2). The decrease in R-squared is greatest when removing BASEDURLOG, followed by PAUSEBIN, and so forth. In sum, variables containing measures obtained by our LDL analysis appear to be meaningful predictors of S duration.

(2) BASEDURLOG > > PAUSEBIN > > COMPONENT3 > > FOLTYPE > > ALC > > DENSITY > > COMPONENT1 > > PREC

**FIGURE 3:**
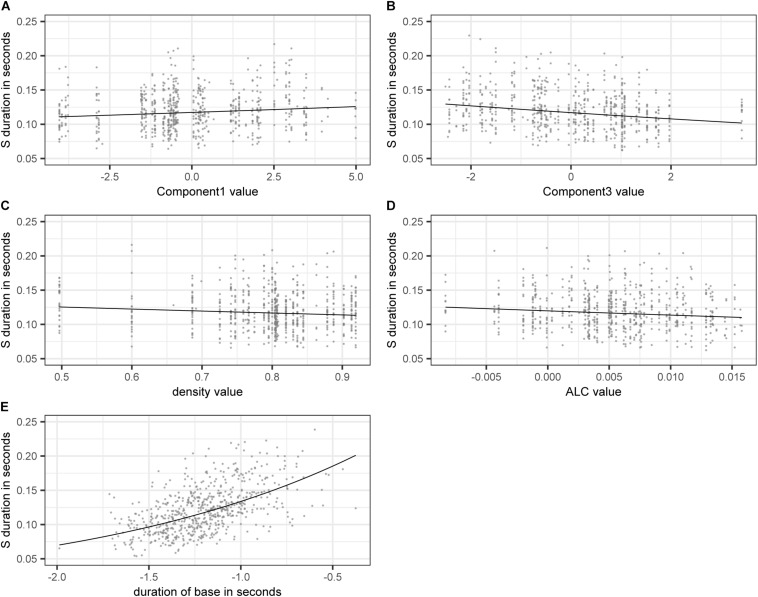
Partial effects of the numerical variables included in the final “LDL measures and AFFIX” model, fitted to the log-transformed values of duration of S. **(A)** COMPONENT1 **(B)** COMPONENT3 **(C)** DENSITY **(D)** ALC **(E)** back-transformed BASEDURLOG.

Figure 3 shows the effect on S duration of the numerical variables included in the model. The estimated values of the dependent variable SDURLOG, i.e., S duration, and BASEDURLOG, i.e., base duration, are back-transformed into seconds. For COMPONENT1, higher values lead to longer S durations, while for COMPONENT3 (panel A), higher values lead to shorter S durations (panel B). Higher values of DENSITY (panel C) and ALC (panel D) come with shorter S durations. Longer bases come with longer S durations (panel E).

**FIGURE 4:**
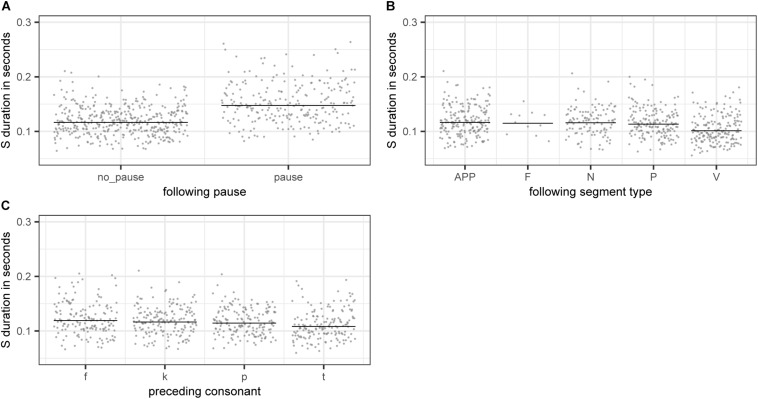
Partial effects of the categorical variables included in the final “LDL measures and AFFIX” model, fitted to the log-transformed values of duration of S. **(A)** PAUSEBIN **(B)** FOLTYPE **(C)** PREC.

The partial effects of the categorical variables included in the final model are illustrated in Figure 4. Pauses lead to longer S durations (panel A), which is most likely a case of phrase-final lengthening (e.g., Cooper and Danly, 1981). There is also an effect of the following segment type, with S being shorter when followed by a vowel (panel B). This difference is significant for all consonant types being compared against vowels with the exception of fricatives. However, as there is only a small number of fricative cases in our data, this non-significant difference is potentially not meaningful. Lastly, there is an effect of preceding consonant on S duration (panel D). S duration is significantly longer if preceded by a voiceless labiodental fricative /f/ or a voiceless velar stop /k/ as compared to cases where S is preceded by a voiceless alveolar stop /t/. All other comparisons are non-significant.

Let us turn to the variables of interest, i.e., those derived from our LDL network. COMPONENT1 acts as a general measure of phonological certainty. High values of COMPONENT1 come with high values of PATH_COUNTS, PATH_ENTROPIES, and ALDC, indicating a high level of phonological uncertainty. At the other end of the COMPONENT1 dimension, high values of PATH_SUM and SUPPORT indicate a high level of phonological certainty. Higher uncertainty appears to lead to longer S durations, while higher certainty appears to lead to shorter S durations.

Recall from Section “Model B: LDL Measures and Affix Specification” that COMPONENT3 relates to semantic activation diversity and to the presence of the plural suffix. Higher values of COMPONENT3 indicate a higher level of semantic activation diversity. Higher levels of activation diversity then lead to shorter S durations (see panel B of Figure 3). High values of COMPONENT3 are positively correlated with the presence of plural S. It appears that the presence of plural makes words semantically more similar to each other as they share this meaning component. Hence it is to be expected that plural words live in a space of greater semantic activation diversity. COMPONENT3 is not only a measure of semantic activation diversity, but also indicates that plural pseudowords show a tendency of having a higher degree of semantic activation diversity as compared to monomorphemic pseudowords in general. DENSITY and ALC also tap into the semantics of pseudowords. That is, similar to COMPONENT3, higher values indicate higher levels of semantic activation diversity. These higher levels then lead to shorter S durations.

### Model C: LDL Measures Only

The final model of LDL measures only is fitted with main effects of the following variables: L1NORM, ALC, NNC, log-transformed base duration (BASEDURLOG), pause (PAUSEBIN), following segmental type (FOLTYPE), and preceding consonant (PREC). The SPEAKER variable is included as random intercept. The p-values of the analysis of variance of the final model are given in Table 8.

**TABLE 8:** *p*-values of fixed effects in the final “LDL measures only” model, fitted to the log-transformed durations of S.

	Sum Sq	Mean Sq	NumDF	DenDF	F.value	Pr (> F)
L1NORM	0.685	0.685	1	611.07	13.473	0.000
BASEDURLOG	6.047	6.047	1	627.51	118.901	0.000
PAUSEBIN	5.440	5.440	1	632.72	106.956	0.000
FOLTYPE	2.056	0.514	4	610.10	10.105	0.000
PREC	0.761	0.254	3	607.96	4.985	0.002
ALC	0.534	0.534	1	615.51	10.504	0.001
NNC	0.778	0.778	1	619.67	15.296	0.000

With a marginal R-squared value of 0.41, the fixed effects of this model explain 41% of variation within the data. The conditional R-squared value of the model is 0.61, that is the complete model accounts for 61% of variation.

The coefficients of the final model and their p-values are given in Table 9. The reference levels for the categorical covariates are: for PAUSEBIN it is no-pause; for FOLTYPE it is APP, and for PREC it is f.

**TABLE 9:** Fixed-effect coefficients and *p*-values as computed by the final “LDL measures” model (mixed-effects model fitted to the log-transformed duration of S).

	Estimate	Std. Error	*df*	*t*-value	Pre (> | t|)
(Intercept)	−2.334	0.320	625.440	−7.301	0.000
L1NORM	−0.044	0.012	611.066	−3.671	0.000
BASEDURLOG	0.624	0.057	627.514	10.904	0.000
PAUSEBINpause	0.233	0.022	632.719	10.342	0.000
FOLTYPEF	−0.019	0.073	613.088	−0.267	0.790
FOLTYPEN	−0.005	0.028	607.324	−0.195	0.845
FOLTYPEP	−0.023	0.024	607.817	−0.950	0.343
FOLTYPEV	−0.140	0.025	611.952	−5.693	0.000
PRECk	−0.029	0.027	607.726	−1.058	0.291
PRECp	−0.053	0.027	607.478	−1.950	0.052
PRECt	−0.101	0.028	608.068	−3.632	0.000
ALC	−6.663	2.056	615.511	−3.241	0.001
NNC	1.221	0.312	619.671	3.911	0.000

As for both other final models, the predictor strength of the individual predictors was checked. Models with one of the predictor variables were constructed based on the complete final model. Then, marginal R-squared values were computed for each of these six models. A comparison of R-squared values then revealed the hierarchy of predictor strength given in (3). That is, the decrease in R-squared is greatest when removing BASEDURLOG, followed by PAUSEBIN, and so forth.

(3) BASEDURLOG > > PAUSEBIN >> FOLTYPE >> NNC >> L1NORM >> ALC >> PREC

Base duration and speaking rate show identical effects as compared to the model fitted in Section “Model B: LDL Measures and AFFIX Specification,” i.e., longer base durations come with longer S durations, while higher speaking rates lead to shorter S durations. As for categorical variables, pauses again come with longer S durations, and S is shorter if followed by a vowel. There is also an effect of the preceding consonant, with S duration being significantly longer if preceded by a voiceless labiodental fricative /f/ or a voiceless velar stop /k/ as compared to cases where S is preceded by a voiceless alveolar stop /t/. These results are generally in line with those by the analysis in the previous section.

**FIGURE 5:**
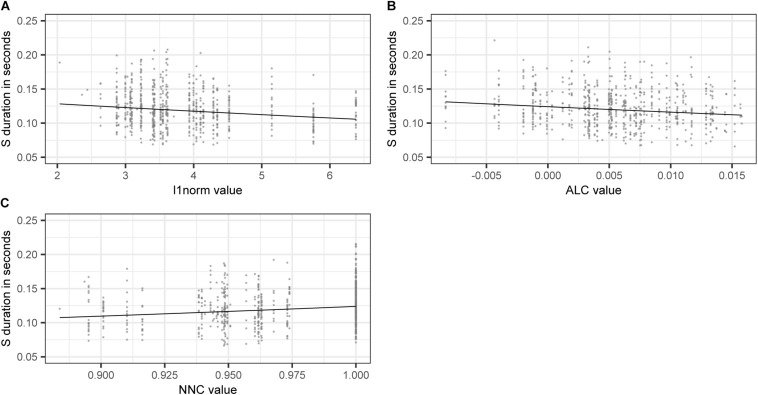
Partial effects of LDL derived variables contained in the final “LDL measures only” model, fitted to the log-transformed values of duration of S. **(A)** L1NORM **(B)** ALC **(C)** NNC.

Taking a closer look at the variables of interest, we find that higher values of L1NORM, and ALC, i.e., higher semantic activation diversity, lead to shorter S durations. As in model B, higher levels of semantic activation diversity come with shorter S durations. For NNC, we find that S duration is longer if a pseudoword is semantically similar to a real word. The effects of L1NORM, ALC, and NNC are illustrated in Figure 5.

## Discussion

### The Present Results

Previous studies (Zimmermann, 2016; Seyfarth et al., 2017; Tomaschek et al., 2019; Plag et al., 2020, 2017; Schmitz et al., 2020) reported that there are significant differences in the acoustic duration between different types of word-final S in English. Such durational differences challenge established feed-forward theories of morphology-phonology interaction (e.g., Chomsky and Halle, 1968; Kiparsky, 1982) as well as theories of psycholinguistics (e.g., Levelt et al., 1999; Roelofs and Ferreira, 2019; Turk and Shattuck-Hufnagel, 2020). The present study investigated whether measures derived on the basis of a discriminative learning theory are predictive of S durations in nonce words. In particular, we implemented LDL networks that model the production of a word based on its relation to the rest of the lexicon.

We explored the predictive possibilities of LDL measures by fitting three different models: a) a model based on the traditional predictors as used in previous studies (Plag et al., 2017; Tomaschek et al., 2019; Schmitz et al., 2020); b) a model with LDL measures and a variable AFFIX specifying the presence or absence of an affix; and c) a model with LDL measures but without a variable specifying the presence or absence of an affix. Both models with LDL measures show that such measures are predictive of S durations. This result is the most important of our study. While traditional variables such as lexical frequencies, bigram frequencies, transitional probabilities or neighbourhood densities measure important lexical properties, it is unclear why they would manifest themselves in a particular morphological effect in speech production. In LDL such effects can emerge through the mapping of form and meaning in a clearly defined process of discriminative learning.

All regression models showed a similar hierarchy of predictor strength for the variables included in the models. For the traditional model A, AFFIX is the third strongest predictor of S duration and for model B this spot is taken by COMPONENT3, while there is no comparable variable included in model C. Comparing the variance explained by the fixed effects of the different models, we find that the traditional model accounts for most variation, i.e., 43%, while the LDL model including the AFFIX variable accounts for 42%, and the LDL model without the AFFIX variable accounts for 41% of variation. Thus, in terms of marginal R-squared values, all three models are close to each other. To check whether these differences in marginal R-squared values are of significance, the three models were refitted to the untrimmed data set and then compared with an analysis of variance. The results suggest that there is no significant difference between the traditional model and the LDL model including the AFFIX variable. However, the LDL model without the AFFIX variable shows a significantly worse fit (*p* < 0.01). This seems to indicate that the LDL measures do not capture the full amount of the variance that is captured by the variable AFFIX. This means that there is still something about the morphological function that translates into duration and that is not properly modelled by the associative measurements of the learning network. The same problem holds, incidentally, for the traditional model (model A), in which the usual lexical measures (such as lexical frequencies, neighbourhood densities, etc.) and phonetic covariates (such as pauses, speech rate, etc.) are also not able to cover all durational variance. The morphological residue in both types of analysis remains a conundrum that calls for more sophisticated approaches in future research.

### Comparison of Results to Other Studies

The LDL measures included in our final models are either concerned with semantic activation diversity (COMPONENT3, ALC, and DENSITY in model B; L1NORM, and ALC in model C), semantic similarity (NNC in model C) or with phonological certainty (COMPONENT1 in model B).

Higher degrees of semantic activation diversity come with shorter S durations. This effect is similar to the one which was reported by Tucker et al. (2019b) in a study on stem vowels, and Tomaschek et al. (2019) in their NDL study on S duration. A higher degree of activation diversity makes it “more difficult to discriminate the targeted outcome from its competitors” (Tomaschek et al., 2019:27). As for production, a prolongation of the acoustic signal is dysfunctional if the prolongation maintains or increases the discrimination problem instead of contributing to resolving it (Tomaschek et al., 2019).

In the model without AFFIX as predictor variable, NNC (i.e., a pseudoword’s semantic similarity to its closest semantic real word neighbour) emerges as significant (see model C). Why so? As reported in Table 2, the AFFIX variable and NNC are strongly negatively correlated (rho = −0.89). Post-hoc analysis shows that plural S has significantly lower NNC values as compared to non-morphemic S (Wilcoxon test, *p* < 0.001). It therefore appears that NNC takes over the role of differentiating between plural and non-morphemic S in model C.

As for phonological certainty, we find that higher phonological certainty leads to shorter S durations, while higher phonological uncertainty leads to longer S durations. Shorter durations in contexts of high phonological certainty may be related to effects of frequency, i.e., highly frequent forms are produced with higher certainty and are thus shorter.

### Directions for Future Research and Conclusion

The results of the present study may bring up further questions. First, are the predictive measures found for word-final S duration in pseudowords also predictive for word-final S duration in real words? Tomaschek et al.’s (2019) NDL implementation suggests that it is, but LDL networks still need to be implemented. It would be especially interesting to model those data sets that have yielded seemingly contradictory effects. Second, taking into account that the specification of AFFIX in the modelling process leads to a significantly better model fit, one may ask what the underlying reasons for this significant effect are. This then automatically leads to another question: Is it possible to catch the effect of the AFFIX specification in terms of (new) LDL measures?

To summarize, this paper was the first to investigate durational differences between different types of word-final S (non-morphemic vs. plural S) in pseudowords by means of an LDL implementation, measures, and resulting statistical analyses. The findings yielded important evidence on the question of how such durational difference come to be, i.e., they can be predicted based on their pseudoword’s relations to the lexicon. We demonstrated that durational differences emerge from the pseudoword’s resonance with the lexicon by way of differing degrees of semantic activation diversity and phonological uncertainty. These manifestations of the relations to other words in the lexicon in turn are the result of discriminative learning.

## Data Availability Statement

The datasets presented in this study can be found in online repositories. The names of the repository/repositories and accession number(s) can be found below: https://osf.io/zy7ar/?view_only=ef43a5caf6444270a56074027d7d6482.

## Author Contributions

DS, IP, and DB-H contributed to conception and design of the study, manuscript revisions. DS retrieved the data and performed the computational implementation supported by SS. DS carried out the modelling and statistical analysis, and wrote the first draft of the manuscript. All authors read and approved the submitted version.

## Conflict of Interest

The authors declare that the research was conducted in the absence of any commercial or financial relationships that could be construed as a potential conflict of interest.

## Publisher’s Note

All claims expressed in this article are solely those of the authors and do not necessarily represent those of their affiliated organizations, or those of the publisher, the editors and the reviewers. Any product that may be evaluated in this article, or claim that may be made by its manufacturer, is not guaranteed or endorsed by the publisher.

## Footnotes

1 The inverse of a matrix needs not exist, rendering such a matrix a singular one. Most matrices used in LDL implementations are singular matrices. Thus, an approximation of the inverse must be used instead of an inverse itself. One such approximation is the Moore-Penrose generalized inverse (Moore, 1920; Penrose, 1955).

2 In addition, a cluster analysis was performed. This analysis revealed clusters which align well with the retained components of the principal component analysis. The cluster analysis is also documented in the materials that can be found at https://osf.io/zy7ar/?view_only=ef43a5caf6444270a56074027d7d6482.
