# Supplementary material for: Durational Differences of Word-Final /s/ Emerge From the Lexicon: Modelling Morpho-Phonetic Effects in Pseudowords With Linear Discriminative Learning
Source: Front Psychol. 2021 Aug 9;12:680889. doi: 10.3389/fpsyg.2021.680889 (PMC8380959; doi:10.3389/fpsyg.2021.680889)
Supplement: Supplementary file 1 [file Table_1.DOCX]

Supplementary Material

Supplementary Table 1. Overview of all pseudowords and their transcriptions used in the current LDL implementation.

| Pseudoword | | Transcription in DISC | Pseudoword | | Transcription in DISC |
| --- | --- | --- | --- | --- | --- |
| blou- | fs | blufs | glai- | fs | gl1fs |
|  | ks | bl{ks; bluks; blVks |  | ks | gl1ks; gl{ks |
|  | ps | blups |  | ps | gl1ps; gl{ps |
|  | ts | bl6ts; bluts |  | ts | gl1ts; gl{ts; gl2ts |
| cloo-fs; -ks; -ps; -ts | | klufs; kluks; klups; kluts | plee-fs; -ks; -ps; -ts | | plifs; pliks; plips; plits |
| gli-fs; -ks; -ps; -ts | | glIfs; glIks; glIps; glIts  glifs; gliks; glips; glits | pru-fs; -ks; -ps; -ts | | prVfs; prVks; prVps; prVts;  prufs; pruks; prups; pruts; |

Supplementary Table 2. Summary of the dependent variable, numerical variables, and categorical variables used in the modelling processes.

| Dependent variable | Mean | | St. Dev. | | Min | | Max | |
| --- | --- | --- | --- | --- | --- | --- | --- | --- |
| sDurLog | -2.116 | | 0.388 | | -3.361 | | -1.221 | |
| Numerical variables | Mean | | St. Dev. | | Min | | Max | |
| speakingRate | 3.607 | | 0.928 | | 1.310 | | 7.100 | |
| baseDurLog | -1.2027 | | 0.232 | | -1.987 | | -0.375 | |
| biphoneProb | 0.001 | | 0.002 | | 0.000 | | 0.004 | |
| age | 28.470 | | 9.323 | | 19.000 | | 58.000 | |
| Component1 | 0.000 | | 1.972 | | -4.036 | | 4.990 | |
| Component2 | 0.000 | | 1.429 | | -3.738 | | 3.752 | |
| Component3 | 0.000 | | 1.273 | | -2.510 | | 3.416 | |
| density | 0.793 | | 0.093 | | 0.497 | | 0.919 | |
| support | 0.880 | | 0.093 | | 0.630 | | 1.011 | |
| ALC | 0.005 | | 0.005 | | -0.008 | | 0.016 | |
| ALDC | 0.710 | | 0.387 | | 0.000 | | 1.400 | |
| EDNN | 0.002 | | 0.002 | | 0.000 | | 0.016 | |
| NNC | 0.970 | | 0.032 | | 0.884 | | 1.000 | |
| Categorical variables | Levels |  | |  | |  | |  |
| affix | NM: 300 | PL: 353 | |  | |  | |  |
| pauseBin | no: 412 | yes: 241 | |  | |  | |  |
| DISC | 38 |  | |  | |  | |  |
| biphoneProbSumBin | high: 161 | low: 492 | |  | |  | |  |
| list | 12 |  | |  | |  | |  |
| slideNumber | 48 |  | |  | |  | |  |
| preC | f: 156 | k: 169 | | p: 164 | | t: 164 | |  |
| folType | APP: 190 | F: 11 | | N: 106 | | P: 165 | | V: 181 |
| speaker | 40 |  | |  | |  | |  |
| gender | 2 |  | |  | |  | |  |
| location | London: 392 | | | elsewhere: 261 | | | |  |
| monoMultilingual | monolingual: 532 | | | bilingual: 121 | | | |  |
| real | FALSE: 542 | TRUE: 111 | |  | |  | |  |
